# Knee stability assessment on anterior cruciate ligament injury: Clinical and biomechanical approaches

**DOI:** 10.1186/1758-2555-1-20

**Published:** 2009-08-27

**Authors:** Mak-Ham Lam, Daniel TP Fong, Patrick SH Yung, Eric PY Ho, Wood-Yee Chan, Kai-Ming Chan

**Affiliations:** 1 Department of Orthopaedics and Traumatology, Prince of Wales Hospital, Faculty of Medicine, The Chinese University of Hong Kong, Hong Kong, PR China; 2 The Hong Kong Jockey Club Sports Medicine and Health Sciences Centre, Faculty of Medicine, The Chinese University of Hong Kong, Hong Kong, PR China; 3 School of Biomedical Sciences, Faculty of Medicine, The Chinese University of Hong Kong, Hong Kong, PR China

## Abstract

Anterior cruciate ligament (ACL) injury is common in knee joint accounting for 40% of sports injury. ACL injury leads to knee instability, therefore, understanding knee stability assessments would be useful for diagnosis of ACL injury, comparison between operation treatments and establishing return-to-sport standard. This article firstly introduces a management model for ACL injury and the contribution of knee stability assessment to the corresponding stages of the model. Secondly, standard clinical examination, intra-operative stability measurement and motion analysis for functional assessment are reviewed. Orthopaedic surgeons and scientists with related background are encouraged to understand knee biomechanics and stability assessment for ACL injury patients.

## Introduction

Sports injury is common, ranking the second highest (21%) in terms of cause of injury [1] and leading to long-term disabilities and handicaps especially in patients with knee injuries [2]. Among all sport-related knee injuries, one-fifth (20%) involves the anterior cruciate ligament (ACL) – the most commonly traumatized structure [3]. ACL rupture results in knee instability [4], prohibits the athletes back to sports, and results in early retirement [5]. Conservative treatments can somewhat enhance the sense of stability and rehabilitation, but not in objective outcome assessment [6] and rate of returning to sports [7]. Therefore, operative treatments are often prescribed to reconstruct the ACL in order to restore the knee stability and return the athletes to sports and active lifestyle [8].

Numerous anatomy studies showed that the intact human ACL consists of an anteromedial (AM) bundle, and a posterolateral (PL) bundle [9], while some studies even reported an intermediate bundle in between [10]. Biomechanics studies showed that AM and PL bundles mainly contribute to anterior-posterior and rotational stability of the knee respectively [11,12]. Traditional surgical methods employ a single bundle bone-patellar-tendon-bone or hamstrings autograph, however, the methods provide good resistance to anterior tibial loads but not to rotational loads [13]. Therefore, the unique anatomical and biomechanics characteristics of the two bundles provide a rationale to the recent emerge of anatomical double-bundle ACL reconstruction approach [14,15] to better mimic and restore the anatomy and biomechanics of the intact ACL in the reconstructed knee [12]. However, this advantage of rotational stability has not been widely proved on living human.

Returning to high level athletic activity is an ultimate goal for patient who undergoes ACL reconstruction. However, standardized and objective criteria to assess athletes' safe return-to-sports are limited. Functional knee stability is proposed to be one of the key factors influencing safe return-to-sports [16]. Before recommending reconstructed patients to return to activity with pre-injury level, good knee stability should be attained when performing similar on-field movements such as stop-jumping and cutting in the laboratory setting. Therefore, functional knee stability evaluated by kinematics assessment definitely provides valuable information on standardization for safe return-to-sports. This article reviews the knee stability assessments for injury diagnosis, treatment evaluation and long term standard for safe return-to-sports for ACL deficient knee. It aims to provide the basic introduction in knee biomechanics and the importance of stability assessments for orthopaedic surgeons, physiotherapists and scientists with related background.

### The knee and its movement

The lower extremity is composed of three major joints: the hip joint, the knee joint and the ankle joint. Located in between hip and ankle joint, knee provides balance and transformation of load of body even when we perform a rapid change of speed and direction. Study has shown that unanticipated cutting maneuvers would increase the risk of non-contact knee ligament injury due to the increased external varus/valgus and internal/external rotation moments applied to the knee [17]. Even in straight running, the ground reaction force can be up to three times the body weight [18]. Therefore, being with the function of supporting the entire body weight during stance phase, knee is one of the most vulnerable joints suffering acute injury [19] and long term development of osteoarthritis [20,21].

#### Anterior cruciate ligament

**Figure 1:**
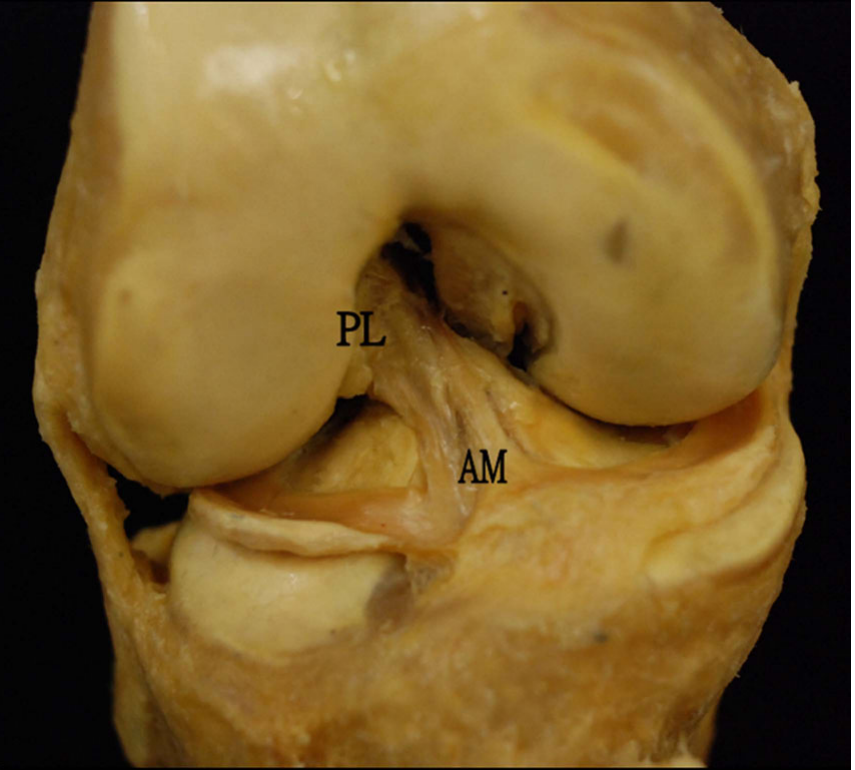
**An anterior view of the right knee, showing anterior cruciate ligament with anteromedial (AM) and posterolateral (PL) bundles**.

The anterior cruciate ligament is a band of dense connective tissue which courses from the femur to the tibia [22]. It is a major knee ligament to stabilize the joint movement against anterior tibial translation [23] and rotational loads [24]. While Norwood and Cross [12] in 1979 suggested ACL to have three separate bundles, most anatomical studies [25,26] agreed that AM bundle and PL bundle are the only two components of ACL (Figure 1). The AM and PL bundles behave differently in length [27] and in situ force [11] during passive flexion. Due to the different bony orientation attachment [27] of the two bundles, AM and PL bundles are responsible for resisting anterior tibial load and rotational load respectively. Biomechanical study [28] revealed the ultimate load of ACL to failure can be as high as three times of the body weight. Video analysis [29] reported that ACL rupture occurs within 100 ms, indicating a huge explosive force acting to the knee joint during ACL injury.

#### Biomechanical presentation of knee motion

The knee joint motion is the relative movement between the femur and the tibia. Theoretically, it is capable to a six degree-of-freedom movement: both translation and rotation in three body planes. Clinically, excessive motion in specific direction (anterior-posterior direction) during physical examination may be an indication of knee ligament injury [30]. The result of these assessments is often determined by the subjective feeling and experience of the examiners. Biomechanical presentation of the knee motion, instead, provides precise information for comparison between intact and deficient knee when assessing knee stability. To describe the geometric representation, Grood and Suntay [31] proposed a joint coordinate system for measuring three dimensional translation and rotation motions of the knee joint. This is essential for studying ligament injury as knee ligaments govern the motion of the knee. For example, ACL rupture would lead to excessive motion in AP translation and tibial rotation [22]. In cadaveric study, it is also suggested that an isolated excision of ACL would increase anterior drawer and tibial rotation in both flexion and extension [26]. Therefore, a well understanding of knee kinematics assessments is crucial for the said purpose of this study.

### Contribution of knee stability assessment at different stages of ACL injury

**Figure 2:**
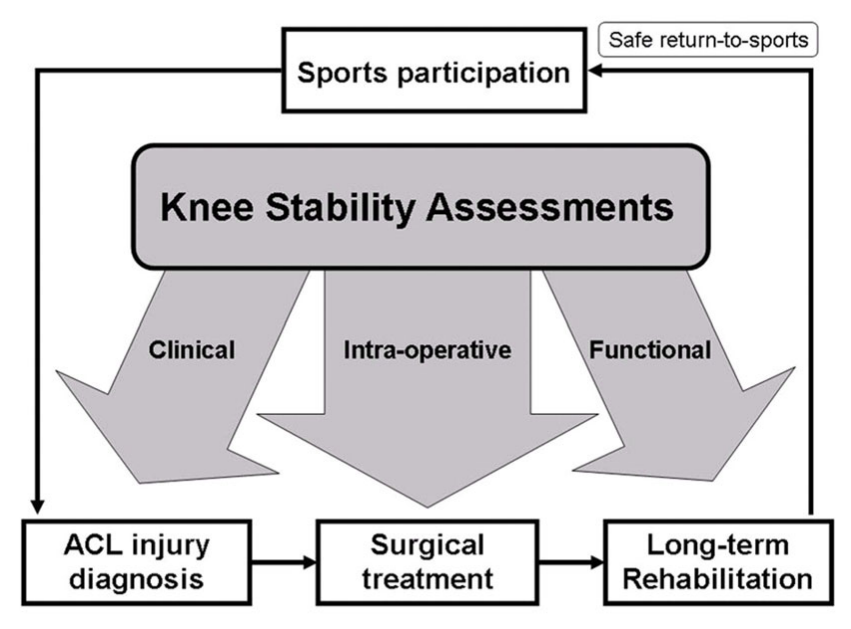
**A management model for ACL injury and contribution of knee stability assessment before safe return to sports**.

Figure 2 shows a management model for ACL injury, starting from sport participation. Most of the ACL injury (approximately 70%) occurs in sport situation [32]. It often appears to occur in competitive sports such as soccer and handball, which involves landing, deceleration and rapid change of direction movements [33]. When injury occurs during sport activity, athlete with ACL rupture is confirmed after an adequate diagnosis by orthopaedic specialist. Either operative or non-operative treatments [34] followed by a rehabilitation program [35] are advised to the injured patients before they can safely return-to-sports [16].

Knee stability assessments contribute three main roles in the management model for ACL injury – (i) clinical assessment provides a quick and reliable way for the diagnosis of ACL injury, (ii) intra-operative assessment evaluates immediate effect of operative treatment and compares different reconstruction techniques, (iii) functional assessment acts as long-term guidelines during or after rehabilitation program, indicating if the athlete is fully recovered in terms of stability to pre-injury activity level. These three main roles are then elaborated in the following sections.

### Diagnosis of anterior cruciate ligament injury – clinical assessment

Accurate diagnosis of ACL injury relies on injury history [36], clinical assessment [37] as well as advanced imaging technique [38]. Being different from others, clinical assessment provides a stability evaluation of the injured knee to test if excessive motion exists. It varies considerably within the normal population and a greater motion would be found in hyper-laxity group [39]. It is always recommended to compare the motion of the injured side to the normal side [40] if the patients have unilateral knee injury. The potential limitations should be kept in mind, including the uncontrolled force applied and the reflex resistance of the patient because of anxiety and pain. The first clinical examination after an acute knee trauma is suggested to have a low diagnostic value [41]. Therefore, clinical assessments should be performed by skillful and experienced examiner. Several typical assessments for diagnosing ACL injury are demonstrated below.

#### Lachman test

**Figure 3:**
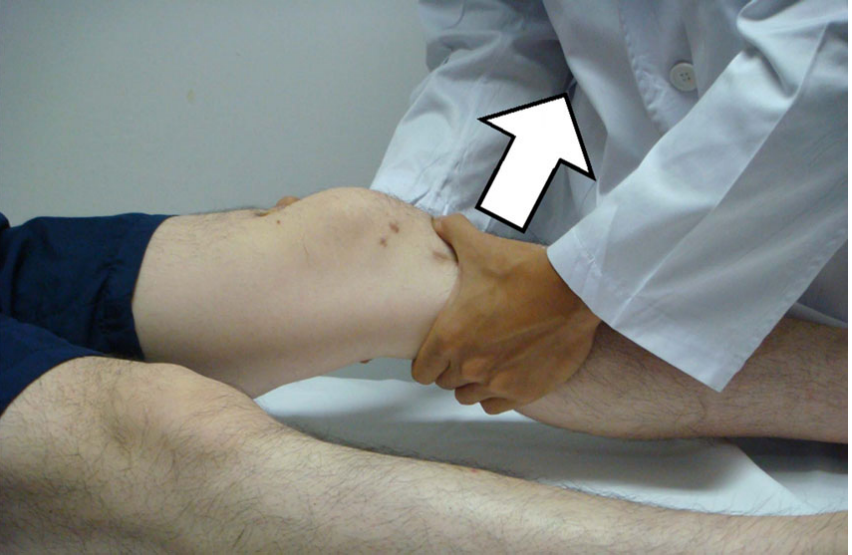
**Lachman test**.

Lachman test has a high accuracy for diagnosing ACL injury [42]. Before the test, the examiner should ensure that the tibia is not subluxated posteriorly to avoid false-positive result in a posterior cruciate ligament deficient knee. The patient is asked to lie supine and the knee flexes around 30°. The examiner stabilizes the femur and applies an anterior force on tibia without restraining axial rotation (Figure 3). A positive result of an ACL deficient knee will be presented with proprioceptive or visible anterior translation of the tibia [43]. The anterior translation of 1 mm to 5 mm is defined as grade I laxity, 6 mm to 10 mm as grade II, and greater than 10 mm or without a displacement limit (end point) as grade III [40]. End point is further graded as firm, marginal or soft.

#### Pivot shift test

**Figure 4:**
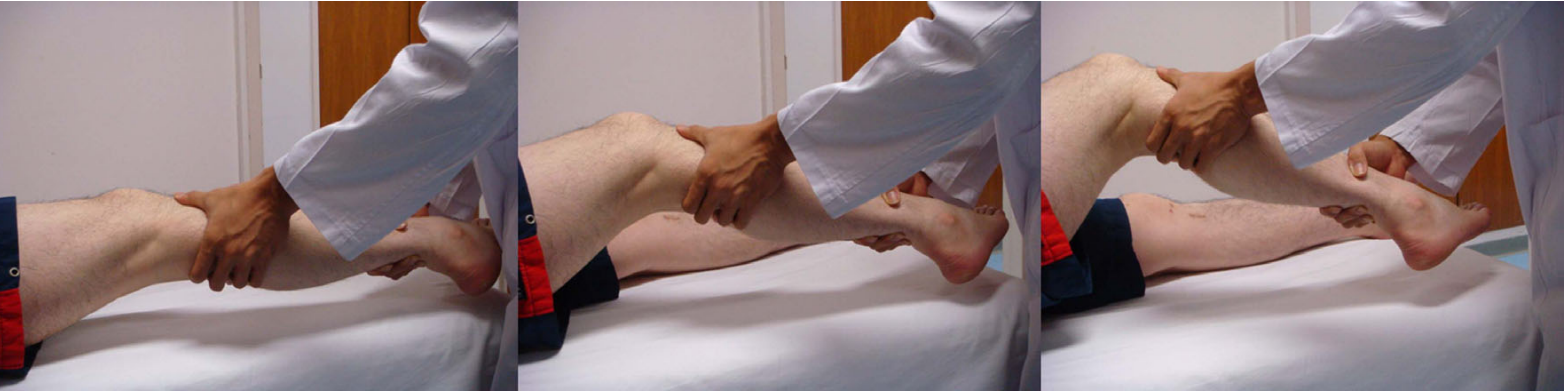
**Pivot shift test**.

Pivot shift test is relatively complex since it is a combination of internal rotation torque and valgus torque [44]. This test is highly dependent to the technique and experience of the examiner as the result is graded subjectively according to the knee laxity. However, a positive result for the pivot shift test is the best for ruling in an ACL rupture [37]. To perform the test, the basic principle is to apply valgus torque and internal rotation to the leg. The test starts with the knee in full extension and then gently flexed to about 40° (Figure 4). A positive pivot shift test is defined as a forward subluxation of tibia during sudden change in direction. It is a reproduction of event that occurs when the knee gives way because of the loss of ACL.

#### KT-1000

**Figure 5:**
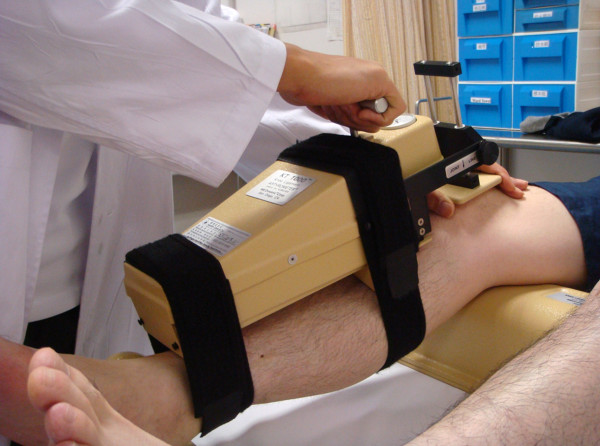
**KT 1000 to measure anterior-posterior laxity of the knee**.

This is an instrument that has been developed for an objective measurement for knee anterior-posterior laxity on sagittal plane. The patient lies in a supine position with knee flexion of about 20° to 30°, supporting with a firm platform placed proximal to the popliteal space. The patient is told to relax in this position. The KT-1000 arthrometer is then placed above the tibia and attached firmly by two bands. After the zero adjustment, the arthrometer is pulled anteriorly to the tibia in order to provide an anterior force (Figure 5). An audible indication will be noticed at 15, 20 and 30 pounds of force. The anterior displacement is measured in millimeter while the laxity is often presented in side-to-side difference.

### Operation treatment evaluation – intra-operative assessment of ACL reconstruction

#### Computer assisted navigation system

Computer-assisted surgery has gone through lots of evolutions in recent 15 years. One of the technologies for orthopaedic surgery is the navigation system, which has been applied in spine surgery [45] and total joint surgery [46]. It has two basic components for ACL reconstruction:

• A set of optical camera to locate the surgical joint and limb, and to create a picture or image of the operation site.

• Computer programs which integrate these images with surgical information and assist the surgeon during the operation.

**Figure 6:**
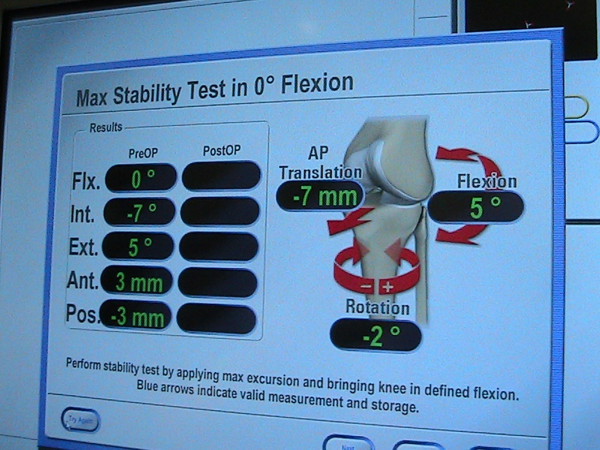
**Kinematics assessment during ACL reconstruction**.

Navigation systems improve the accuracy of surgical procedures [47]. The computer provides information of the real time relative positions between the instruments and the bone to assist surgeon during surgical procedures. Moreover, by locating joint centre between two relative bodies, it accurately measures the kinematics data in sagittal, coronal or transverse plane (Figure 6). This technique has been utilized in the total knee replacement surgery to guide the balancing of ligaments [46]. In the same way, computer assisted navigation system is employed to collect intra-operative details on the laxity of knee in different planes both before and after the ACL reconstruction [48]. Therefore, it would be a good way to assess immediate effect of ACL reconstruction, especially to compare single-bundle technique and anatomical double-bundle technique in terms of anterior translation and tibial rotation.

#### Navigation details and measurement

**Figure 7:**
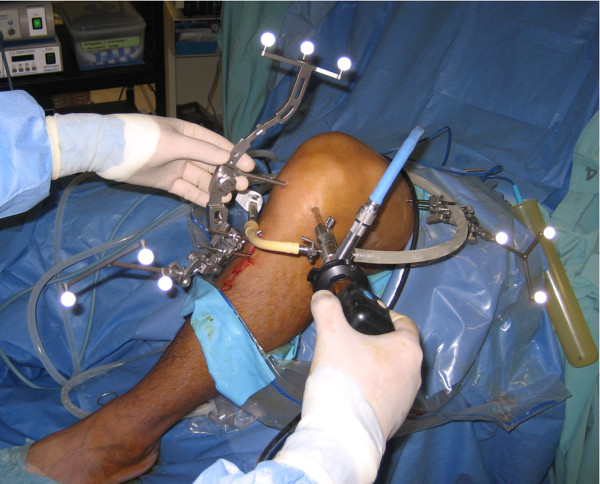
**Femoral and tibial transmitters are inserted into bone during navigation process**.

Fluoroscopic navigation system [47,49] is based on the pre imaging data (both AP and lateral views), such as computer tomography or C-arm shots, for the model formation to be displaced in the computer software. A pointer containing integrated reflective markers discs is also attached to the C-arm image. By holding the pointer to the known anatomical landmarks, the surgeon reviews the accuracy of the images when acquiring the AP and lateral images. To accurately locate the navigated tools in relationship to the selected anatomic landmarks, surgical instrument with passive marker spheres must be fixed securely to the patient's femur and tibia (Figure 7). Optoelectronic camera system with infrared light-emitting diodes tracks all passive markers throughout the surgical procedure. The line of sight must be guaranteed once after the navigated procedure starts.

Image-free navigation [50] has been widely established recently. Based on the intra-operative knee alignment measurement such as knee axis and joint lines, the system provides virtual illustration of the anatomical structures. With the information after digitizing the cartilage surface of the femur and the tibia, this method combines the existing model and patient's knee as defined by surface matching.

For both fluoroscopic and image-free navigations, since one reference base is fixed each to the femur and the tibia the relative motion in space can be measured precisely. A few studies have reported the validity and reliability of the navigation system. Tsuda et al. [50] validated the navigation system for femoral tunnel placement in double-bundle ACL reconstruction with motion analysis system (digital camera). The average differences between the two measurement systems were less than 3% for both AM and PL tunnels. Another studies conducted by Martelli et al. [51], Pearle et al. [52] and Colombet et al. [53] reported that navigation system is reliable to quantify knee kinematics during stability examinations, particularly in the setting of complex rotatory patterns such as pivot shift test. This suggests an accurate and precise evaluation of different techniques of ACL reconstruction.

### Long term evaluation during and after rehabilitation – functional assessment

#### Passive and active motion

By applying a certain force on specific direction to the relaxed knee, ligament injury would be identified if laxity is found when comparing to the other side. This is a usual clinical examination for suspected knee injury without any patients' active movement. However, it may not be the best assessment when it comes to the rehabilitation stage after operative treatment as clinical examinations do not produce sufficient force to stimulate physical activity [40]. The ultimate goal for clinical treatment in sports medicine is to allow patients' safe return-to-sports. It was also suggested that functional knee stability should be one of the criteria that determine a safe return-to-sports [16]. Being different from static knee stability test such as KT-1000, dynamic functional test, which mimics real game situation during sports, involves patients' muscle strength and neuromuscular perception, demand of specific movement and confidence for performing. To monitor the knee stability during this specific dynamic movement, motion analysis is a good way to achieve.

#### Optical motion analysis with reflective skin marker

**Figure 8:**
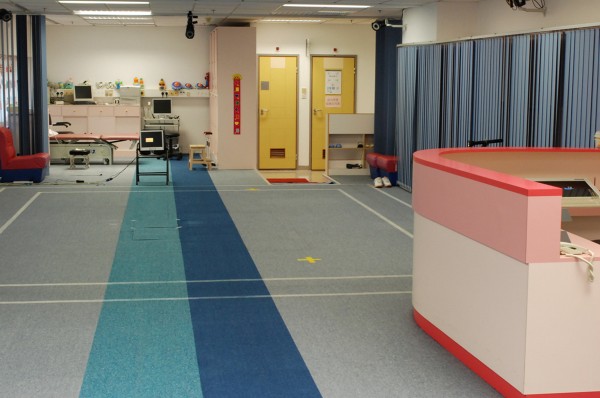
**Gait Laboratory**.

Patients with ACL injury can be assessed using motion analysis before and after ACL reconstruction. The functional assessment is conducted in a gait laboratory (Figure 8), which is equipped of more than three high-speed cameras and data processing software, providing 10 × 5 m2 captured volume. The three dimensional coordinates of nine-millimeter reflective markers can be recognized in the captured volume by means of infra-red light emitting cameras. In the centre of the captured volume, force plates are placed on floor level in order to collect ground reaction force during the movement.

**Figure 9:**
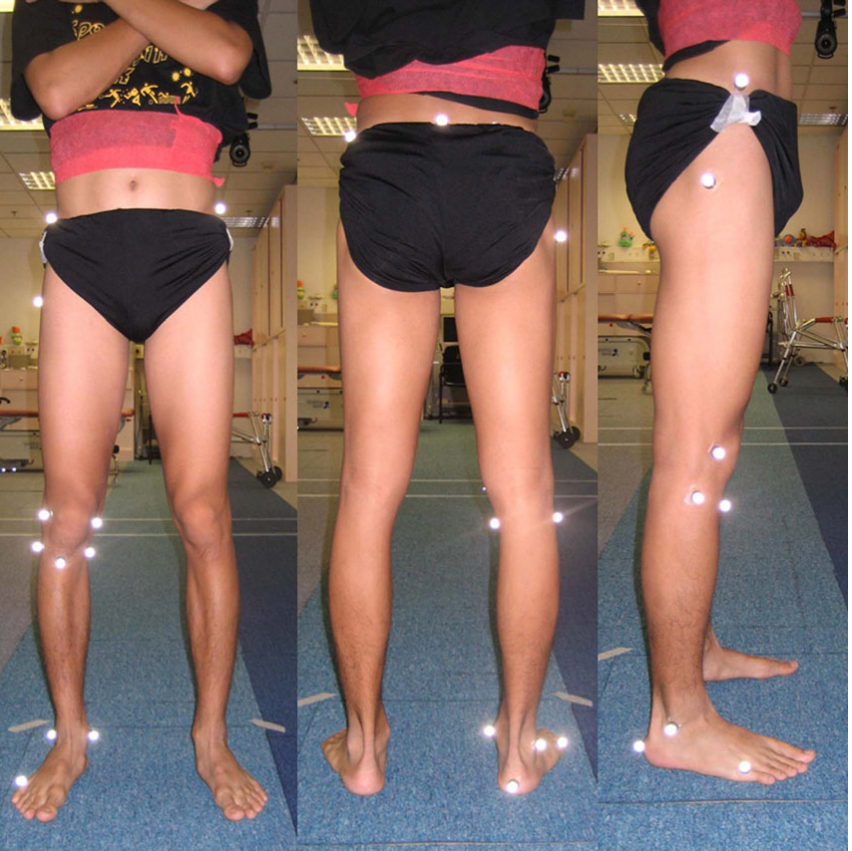
**Marker set of motion analysis assessment (Left to right: anterior view, posterior view and lateral view)**.

Marker model is essential for motion analysis. It consists of several reflective skin markers that depend on outcome parameters. Ristanis et al [54] adopt the method described by Vaughan [55] for measuring knee joint kinematics. Fifteen markers are stuck on anatomical landmarks of lower extremities including anterior superior iliac spine (ASIS), greater trochanter, lateral femoral epicondyle, tibial tubercle, lateral malleolus, heel, metatarsal head V on both sides and sacrum (Figure 9). Before capturing the dynamic movement, anthropometric data which include weight, ASIS breadth and thigh length, medthigh circumference, calf length, calf circumference, knee diameter, foot length, malleolus height, malleolus diameter, foot breadth on both sides, are collected.

After data collection, the evaluation period should be well defined and trimmed. In clinical practice, stance phase is chosen for evaluation due to the landing risk factor of noncontact ACL injury. A standing trial with anatomical position is needed to define the offset degree for all segmental movements in all planes. Kinematics of knee joint such as flexion angle, tibial rotation and valgus angle are calculated using programming software. Anthropometric measurements combined with three dimensional coordinates from standing trial provide joint centre position and axes of joint rotations. Joint kinematics is then calculated from the position of reflective markers during the movements.

#### The dynamic movement

Dynamic movement should be clinically based and specific to the research objective. ACL injury would lead translational and rotational instability. The movement that performed by the ACL deficient and reconstructed patients should be high demanding, giving a rotational and valgus stress to the knee. Ristanis et al [54] employ a combined movement in assessing ACL deficient and reconstructed patients. The movement involves jumping, landing, pivoting and running. The patient is required to jump forward from a 40 cm high platform, land with both feet, pivot to the right or left at 90° and run away with their maximum speed. It is treated as a high demand of activity in which the movement has to resist a high rotational stress to the knee during pivoting.

**Figure 10:**
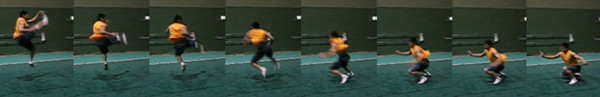
**Non-contact anterior cruciate ligament injury**.

The injury mechanism can be an implication of how we assess ACL injury patients. It is reported that over 70% of ACL injury occur in non-contact situation (Figure 10), which involves landing, decelerating and changing direction [33]. If the patient has good stability during these 'high risk' movements, it would be an important indication for safe return-to-sports. Therefore, movements such as 3-way cutting [56] and 4-way jumping [57] maneuvers are useful to assess the knee stability. In the cutting task, patients are required to run and cut with single leg in three directions: 90° cut, 45° cut and crossover cut. The 4-way jumping task consists of straight run and a two-footed landing followed by a two-footed takeoff maximum jumping in four directions: vertical, anterior, right and left. After data collection, stance phase is trimmed for further evaluation. Kinematics and kinetics data will be measured from the motion analysis system and the force-plate. Both injury (deficient or reconstructed) and intact knee should be assessed since comparison is important when investigating knee laxity.

In most situations during sports, movements such as landing and sudden change of direction are often unexpected. Planned laboratory experiments and actual athletic competition would result different biomechanics performances [17]. Biomechanics study has also shown that unplanned cutting is identified as a risk factor of noncontact ACL injury [58]. In order to investigate this unanticipated effect, a device containing photo cell receiver with light source is instrumented across the runway. When the patient passes through the device, a randomized signal will be generated from the computer connecting to the device. It will then create a visual cue for the cutting and jumping direction through a monitor placing in front of the patient This laboratory setting would only allow subjects' short time decision so that a game-like situation is reproduced in the laboratory.

## Discussion

Standard clinical tests, such as Anterior Drawer test and Lachman test, are commonly used to assess AP stability before and after reconstructing the graft. With the help of validated navigation system, knee kinematics stability test can be assessed during operation procedure, enabling the evaluation of immediate effect of ACL reconstruction. The clinical result in terms of laxity is more reliable using navigation system when compared to conventional procedure [59]. To investigate if ACL reconstruction with anatomical double-bundle technique better improve rotational stability, Robinson et al [60] suggested that PL bundle was important than AM bundle in controlling rotational component during Pivot Shift test. In another intra-operative study [61] in which the surgeon applied manual maximum force to test anterior-posterior and rotational stability, however, found no significant different between single-bundle technique and double-bundle technique in restoring knee kinematics. It is still a controversial issue for double-bundle technique before it comes to a consensus from different research groups.

Patients with ACL deficiency report that they feel giving way rather than anterior-posterior instability during cutting movement in sports. Pivot Shift test is a dynamic test containing multiple directional motion to assess abnormal joint excursion [53]. Using navigation system, stability in terms of rotational displacement and anterior translation can be objectively monitored during Pivot Shift test. However, the manual force applied by the surgeon remains one of major limitations in these intra-operative studies [53,60,61]. Robotic testing systems have been employed in cadaveric experiments to simulate Pivot Shift test to a combined valgus and internal rotatory loads [44,62]. This kind of equipments with controlled manual force should be implemented to the operation theatre for future study which aims at a more scientific proof for having double-bundle technique on ACL injury patients.

**Figure 11:**
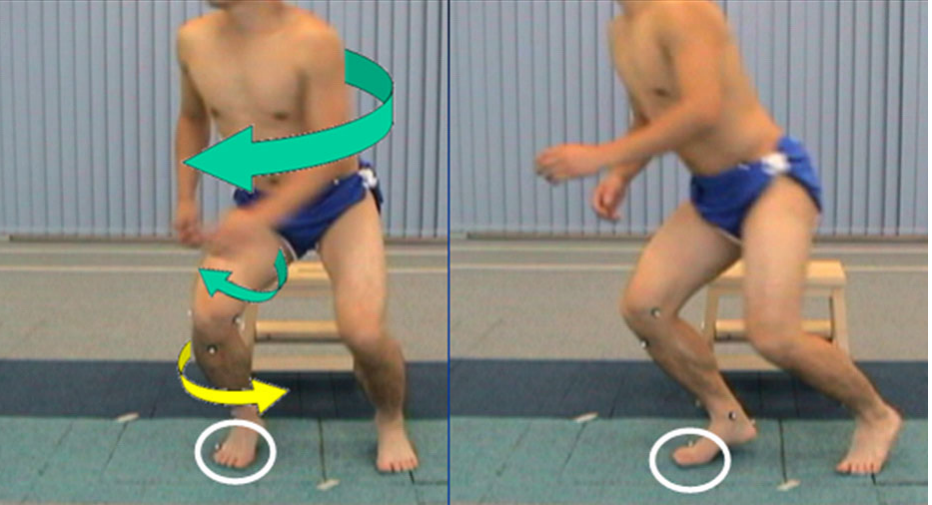
**Rotation mechanism during pivoting movement: The upper body and femur externally rotate during the start of pivoting**. While the fore foot is sticking on the ground, the tibia internally rotates as a result.

For the dynamic pivoting movement, the evaluation period is identified during the stance phase of the pivoting knee, from the first contact of landing to the take-off after pivoting. Knee joint kinematics should be focused during the pivoting movement as it gives a high rotational stress on the knee. When it starts to pivot, the upper body with the femur will externally rotate. Meanwhile, the fore foot of the pivoting leg is sticking on the ground, the tibia then internally rotates relatively to a maximum point as a result (Figure 11).

In the study conducted by Ristanis et al [54], the range of internal rotation was reported to be significantly higher in deficient knees than that in intact knees. The authors, however, did not mention about the other knee kinematics data such as valgus angle during the landing phase, which might be an important implication of instability of ACL deficient patients. This kinematics study maneuver, which demonstrates a similar clinical result [63], not only further confirms the rotational laxity in ACL deficient patients, but also provides an adequate assessment for the long term evaluation of anatomical double-bundle ACL reconstruction.

## Conclusion

The knee stability assessments in different stages of management model for ACL injury are important in sports medicine. Related researches on clinical examination, intra-operative navigation ACL reconstruction and functional evaluation with motion analysis system are highlighted for better understanding of how these assessments contribute to the diagnosis of ACL injury, the immediate evaluation of operation treatments and the establishment of safe return-to-sports criteria respectively. The clinical relevance is for orthopaedic surgeons, physiotherapists and scientists with related background to apply appropriate assessments for ACL injury patients.

## Competing interests

There are no sources of funding used to assist in the preparation of this manuscript.

There are no potential conflicts of interest the authors may have that are relevant to the contents of this manuscript.

## Authors' contributions

MHL drafted the manuscript, PSHY participated in study design and manuscript structure, EPYH advised clinical opinions for assessing stability, DTPF advised on biomechanical assessment and drafted the manuscript, WYC provided pictures of cadaver specimens and advised on anatomical knowledge, KMC provided equipments and clinical setting. All authors read and approved the final manuscript.

## References

[B1] Dekker R, Kingma J, Groothoff JW, Eisma WH, Ten Duis HJ (2000). Measurement of severity of sports injuries: an epidemiological study. Clin Rehabil.

[B2] Dekker R, Groothoff J, Sluis C Van der, Eisma W, Ten Duis H (2003). Long-term disabilities and handicaps following sports injuries: outcome after outpatient treatment. Disabil Rehabil.

[B3] Majewski M, Susanne H, Klaus S (2006). Epidemiology of athletic knee injuries: A 10-year study. Knee Surg Sport Tr A.

[B4] Veltri DM, Deng XH, Torzilli PA, Warren RF, Maynard MJ (1995). The role of the cruciate and posterolateral ligaments in stability of the knee. A biomechanical study. Am J Sports Med.

[B5] Myklebust G, Bahr R (2005). Return to play guidelines after anterior cruciate ligament surgery. Brit J Sport Med.

[B6] Swirtun LR, Jansson A, Renstrom P (2005). The effects of a functional knee brace during early treatment of patients with a nonoperated acute anterior cruciate ligament tear: a prospective randomized study. Clin J Sport Med.

[B7] Fink C, Hoser C, Hackl W, Navarro RA, Benedetto KP (2001). Long-term outcome of operative or nonoperative treatment of anterior cruciate ligament rupture – is sports activity a determining variable?. Int J Sports Med.

[B8] Gotlin RS, Huie G (2000). Anterior cruciate ligament injuries. Operative and rehabilitative options. Phys Med Rehabil Clin N Am.

[B9] Petersen W, Zantop T (2007). Anatomy of the anterior cruciate ligament with regard to its two bundles. Clin Orthop Relat R.

[B10] Zaricznyj B (1987). Reconstruction of the anterior cruciate ligament of the knee using a doubled tendon graft. Clin Orthop Relat R.

[B11] Gabriel MT, Wong EK, Woo SL, Yagi M, Debski RE (2004). Distribution of in situ forces in the anterior cruciate ligament in response to rotatory loads. J Orthopaed Res.

[B12] Norwood LA, Cross MJ (1979). Anterior cruciate ligament: functional anatomy of its bundles in rotatory instabilities. Am J Sport Med.

[B13] Woo SL, Kanamori A, Zeminski J, Yagi M, Papageorgiou C, Fu FH (2002). The effectiveness of reconstruction of the anterior cruciate ligament with hamstrings and patellar tendon. A cadaveric study comparing anterior tibial and rotational loads. J Bone Joint Surg Am.

[B14] Hara K, Kubo T, Suginoshita T, Shimizu C, Hirasawa Y (2000). Reconstruction of the anterior cruciate ligament using a double bundle. Arthroscopy.

[B15] Yagi M, Kuroda R, Nagamune K, Yoshiya S, Kurosaka M (2007). Double-bundle ACL reconstruction can improve rotational stability. Clin Orthop Relat R.

[B16] Kvist J (2004). Rehabilitation following anterior cruciate ligament injury: current recommendations for sports participation. Sports Med.

[B17] Besier T, Lloyd D, Ackland T, Timothy R, Cochrane J (2001). Anticipatory effects on knee joint loading during running and cutting maneuvers. Med Sci Sport Exer.

[B18] Cavanagh P, Lafortune M (1980). Ground reaction forces in distance running. J Biomech.

[B19] Adirim TA, Cheng TL (2003). Overview of injuries in the young athlete. Sports Med.

[B20] Drawer S, Fuller CW (2001). Propensity for osteoarthritis and lower limb joint pain in retired professional soccer players. Brit J Sport Med.

[B21] DeHaven KE, Cosgarea AJ, Sebastianelli WJ (2003). Arthrofibrosis of the knee following ligament surgery. Instr Course Lect.

[B22] Duthon VB, Barea C, Abrassart S, Fasel JH, Fritschy D, Menetrey J (2006). Anatomy of the anterior cruciate ligament. Knee Surg Sport Tr A.

[B23] Furman W, Marshall JL, Girgis FG (1976). The anterior cruciate ligament – A functional analysis based on postmortem studies. J Bone Joint Surg Am.

[B24] Fu FH, Zelle BA (2007). Rotational instability of the knee: editorial comment. Clin Orthop Relat R.

[B25] Lam SJ (1968). Reconstruction of the anterior cruciate ligament using the Jones Procedure and its Guy's Hospital Modification. J Bone Joint Surg Am.

[B26] Girgis FG, Marshall JL, Monajem A (1975). The cruciate ligaments of the knee joint. Anatomical, functional and experimental analysis. Clin Orthop Relat R.

[B27] Hollis JM, Takai S, Adams DJ, Horibe S, Woo SL (1991). The effects of knee motion and external loading on the length of the anterior cruciate ligament (ACL): a kinematic study. J Biomed Eng.

[B28] Woo SL, Hollis JM, Adams DJ, Lyon RM, Takai S (1991). Tensile properties of the human femur-anterior cruciate ligament-tibia complex. The effects of specimen age and orientation. Am J Sports Med.

[B29] Krosshaug T, Slauterbeck JR, Engebretsen L, Bahr R (2007). Biomechanical analysis of anterior cruciate ligament injury mechanisms: three-dimensional motion reconstruction from video sequences. Scand J Med Sci Spor.

[B30] Woo S, Debski R, Withrow J, Janaushek M (1999). Biomechanics of knee ligaments. Am J Sport Med.

[B31] Grood E, Suntay W (1983). A joint coordinate system for the clinical description of three-dimensional motions: application to the knee. J Biomed Eng.

[B32] Smith BA, Livesay GA, Woo SL (1993). Biology and biomechanics of the anterior cruciate ligament. Clin Sport Med.

[B33] Hughes G, Watkins J (2006). A risk-factor model for anterior cruciate ligament injury. Sports Med.

[B34] Beynnon BD, Johnson RJ, Abate JA, Fleming BC, Nichols CE (2005). Treatment of anterior cruciate ligament injuries, part I. Am J Sport Med.

[B35] Myer GD, Paterno MV, Ford KR, Quatman CE, Hewett TE (2006). Rehabilitation after anterior cruciate ligament reconstruction: criteria-based progression through the return-to-sport phase. J Orthop Sport Phys.

[B36] Krosshaug T, Andersen TE, Olsen OE, Myklebust G, Bahr R (2005). Research approaches to describe the mechanisms of injuries in sport: limitations and possibilities. Brit J Sport Med.

[B37] Ostrowski JA (2006). Accuracy of 3 diagnostic tests for anterior cruciate ligament tears. J Athl Training.

[B38] Klass D, Toms AP, Greenwood R, Hopgood P (2007). MR imaging of acute anterior cruciate ligament injuries. Knee.

[B39] Renstrom P, Ljungqvist A, Arendt E, Beynnon B, Fukubayashi T, Garrett W, Georgoulis T, Hewett TE, Johnson R, Krosshaug T, Mandelbaum B, Micheli L, Myklebust G, Roos E, Roos H, Schamasch P, Shultz S, Werner S, Wojtys E, Engebretsen L (2008). Non-contact ACL injuries in female athletes: an International Olympic Committee current concepts statement. Br J Sports Med.

[B40] Lubowitz JH, Bernardini BJ, Reid JB (2008). Current concepts review: comprehensive physical examination for instability of the knee. Am J Sport Med.

[B41] Frobell RB, Lohmander LS, Roos HP (2007). Acute rotational trauma to the knee: poor agreement between clinical assessment and magnetic resonance imaging findings. Scand J Med Sci Spor.

[B42] Jonsson T, Althoff B, Peterson L, Renstrom P (1982). Clinical diagnosis of ruptures of the anterior cruciate ligament: a comparative study of the Lachman test and the anterior drawer sign. Am J Sports Med.

[B43] Torg JS, Conrad W, Kalen V (1976). Clinical diagnosis of anterior cruciate ligament instability in the athlete. Am J Sports Med.

[B44] Kanamori A, Zeminski J, Rudy TW, Li G, Fu FH, Woo SL (2002). The effect of axial tibial torque on the function of the anterior cruciate ligament: a biomechanical study of a simulated pivot shift test. Arthroscopy.

[B45] Holly L, Foley K (2003). Intraoperative spinal navigation. Spine.

[B46] Laskin R, Beksac B (2006). Computer-assisted navigation in TKA: where we are and where we are going. Clin Orthop Relat R.

[B47] Hufner T, Meller R, Kendoff D, Zeichen J, Zelle BA, Fu FH, Krettek C (2005). The role of navigation in knee surgery and evaluation of three-dimensional knee kinematics. Oper Techn Orthopaedics.

[B48] Koh J (2005). Computer-assisted navigation and anterior cruciate ligament reconstruction: accuracy and outcomes. Orthopedics.

[B49] Shafizadeh S, Huver HJ, Grote S, Hoeher J, Paffrath T, Tiling T, Bouillon B (2005). Principles of fluoroscopic-based navigation in anterior cruciate ligament reconstruction. Oper Techn Orthopaedics.

[B50] Tsuda E, Ishibashi Y, Fukuda A, Tsukada H, Toh S (2007). Validation of computer-assisted double-bundle anterior cruciate ligament reconstruction. Orthopedics.

[B51] Martelli S, Zaffagnini S, Bignozzi S, Bontempi M, Marcacci M (2006). Validation of a new protocol for computer-assisted evaluation of kinematics of double-bundle ACL reconstruction. Clin Biomech.

[B52] Pearle AD, Solomon DJ, Wanich T, Moreau-Gaudry A, Granchi CC, Wickiewicz TL, Warren RF (2007). Reliability of navigated knee stability examination: a cadaveric evaluation. Am J Sport Med.

[B53] Colombet P, Robinson J, Christel P, Franceschi JP, Djian P (2007). Using navigation to measure rotation kinematics during ACL reconstruction. Clin Orthop Relat R.

[B54] Ristanis S, Giakas G, Papageorgiou CD, Moraiti T, Stergiou N, Georgoulis AD (2003). The effects of anterior cruciate ligament reconstruction on tibial rotation during pivoting after descending stairs. Knee Surg Sport Tr A.

[B55] Vaughan C, Davis B, O'Conner J (1992). Dynamics of Human Gait.

[B56] Waite JC, Beard DJ, Dodd CA, Murray DW, Gill HS (2005). In vivo kinematics of the ACL-deficient limb during running and cutting. Knee Surg Sport Tr A.

[B57] Sell TC, Ferris CM, Abt JP, Tsai YS, Myers JB, Fu FH, Lephart SM (2006). The effect of direction and reaction on the neuromuscular and biomechanical characteristics of the knee during tasks that simulate the noncontact anterior cruciate ligament injury mechanism. Am J Sport Med.

[B58] Landry S, McKean K, Hubley-Kozey C (2007). Neuromuscular and lower limb biomechanical differences exist between male and female elite adolescent soccer players during an unanticipated side-cut maneuver. Am J Sport Med.

[B59] Plaweski S, Cazal J, Rosell P, Merloz P (2006). Anterior cruciate ligament reconstruction using navigation – A comparative study on 60 patients. Am J Sport Med.

[B60] Robinson J, Carrat L, Granchi C, Colombet P (2007). Influence of anterior cruciate ligament bundles on knee kinematics: clinical assessment using computer-assisted navigation. Am J Sport Med.

[B61] Ferretti A, Monaco E, Labianca L, Conteduca F, De Carli A (2008). Double-bundle anterior cruciate ligament reconstruction: a computer-assisted orthopaedic surgery study [see comment]. Am J Sport Med.

[B62] Bull AM, Andersen HN, Basso O, Targett J, Amis AA (1999). Incidence and mechanism of the pivot shift. An in vitro study. Clin Orthop Relat R.

[B63] Siebold R, Dehler C, Ellert T (2008). Prospective randomized comparison of double-bundle versus single-bundle anterior cruciate ligament reconstruction. Arthroscopy.

